# Resveratrol effects in bladder cancer: A mini review

**DOI:** 10.1590/1678-4685-GMB-2020-0371

**Published:** 2021-03-22

**Authors:** Tamires Cunha Almeida, Glenda Nicioli da Silva

**Affiliations:** 1 Universidade Federal de Ouro Preto, Laboratório de Pesquisas Clínicas, Ouro Preto, MG, Brazil.

**Keywords:** Apoptosis, bladder cancer, cell cycle arrest, cell signalling, resveratrol

## Abstract

Bladder cancer has a high incidence worldwide and is the most common genitourinary cancer. The treatment of bladder cancer involves surgery and chemotherapy; however high failure rates and toxicity are observed. In this context, the search of new drugs aiming a more effective treatment is extremely necessary. Natural products are an important source of compounds with antiproliferative effects. Resveratrol is a naturally occurring plant polyphenol whose anticancer activity has been demonstrated in different types of cancer. This review summarizes the *in vitro* and *in vivo* studies using models of bladder cancer treated with resveratrol and discusses its different mechanisms of action.

## Introduction

Bladder cancer is the most common tumor of the urinary system (Siegel *et al*., 2018), with approximately 550,000 new cases every year (Richters *et al*., 2019). Although the highest incidence rates occur in North America, Europe and parts of Western Asia, the mortality rates are greater on developing areas (Dy *et al*., 2017). The diagnosis occurs predominantly after the age of 55 and the detection of bladder cancer in children and young adults is rare (Saginala *et al*., 2020). Tobacco and occupational exposure to aromatic amines and polyaromatic hydrocarbons are the main risk factors (Cumberbatch *et al*., 2018).

Transitional cell carcinoma, also called urothelial carcinoma, is the most common histological type and comprises more than 90% of bladder cancers. Other cell types include squamous cell carcinoma, adenocarcinoma and small-cell carcinoma (Hoskin and Dubash, 2012).

Superficial bladder cancers, confined to the bladder mucosa or submucosal layer, are managed with resection and intravesical therapy. In contrast, muscle invasive bladder cancers are treated with more aggressive procedures, as partial or total cystectomy, with or without chemotherapy (Sanli *et al*., 2017). Unfortunately, therapeutic failure can occur as lack of drug efficacy, occurrence of serious adverse effects or tumoral progression and recurrence. For example, approximately half of the patients with superficial bladder cancer fail to respond to intravesical bacillus Calmette-Guérin treatment and have a greater chance to progress to muscle invasive disease or present recurrence (Shiota *et al*., 2020). In the chemotherapy before radical cystectomy, approximately half of the patients do not respond to cisplatin-based chemotherapy and can be affected by toxic side effects (Funt and Rosenberg, 2017).

Although the use of traditional medicine is less frequent (Oyebode *et al*., 2016), the search of new drugs from natural sources is still of great importance. From 1940 to 2014, approximately 49% of molecules approved to cancer chemotherapy are derived from natural products (Newman and Cragg, 2016).

Resveratrol (RSV) is a polyphenolic compound found in grapes, blackberries, blueberries, raspberries and peanuts. A widely known source of resveratrol is red wine, which contain resveratrol concentrations from 1.9-14.3 mg/L, depending on grape variety, cultivation place and preparation method (Stephan *et al*., 2017). However, the dominant natural source of RSV is *Polygonum cuspidatum*, which is extensively used in traditional Chinese and Japanese medicine (Liu *et al*., 2019). *P. cuspidatum* leaves present 1000 µg/g of RSV (Liu *et al*., 2019). Li X *et al.* (2006) observed extremely high extractable amounts of RSV in berry skins [>100 µg/g of skin fresh weight (FW)] and seeds (>20 µg/g of seed FW) in two rootstock cultivars obtained from hybrids of V. *monticula* × V. *riparia*. The authors also showed red-berry cultivars had significantly higher amounts of extractable RSV in skin and seeds (0.66-1.44 µg/g of skin FW and 1.34-1.40 µg/g of seed FW) than green-berry cultivars (0.44-0.73 µg/g of skin FW and 1.22-1.23 µg/g of seed FW). Moreover, the RSV concentration in peanuts is about 1.9 µg/g (Sales and Resurreccion, 2014).

RSV presents numerous biological activities, such as cardioprotective (Wu and Hsieh, 2011), antioxidant (Carrizzo *et al*., 2013), anti-inflammatory (de Sá Coutinho *et al*., 2018), antibacterial and antifungal (Vestergaard and Ingmer, 2019), anti-aging (Li Y *et al*., 2018), neuroprotective (Bastianetto *et al*., 2015), and others. Jang *et al.* (1997) were the first to demonstrate the antitumor properties of RSV on the three stages of the carcinogenesis process. Over the years, RSV effects on different types of cancer have been demonstrated and several reviews have been published about these findings (Sinha *et al*., 2016; Yousef *et al*., 2017; De Amicis *et al*., 2019; Huang *et al*., 2019). Moreover, the selectivity of resveratrol for tumor cells compared to normal cells (immortalized SV-HUC-1 normal human urothelial cells) has already been demonstrated (Zhou *et al*., 2014). Here, we focus on summarizing the *in vitro* and *in vivo* studies that used RSV on bladder cancer models. To the best of our knowledge, this is the first review that summarizes studies about RSV and bladder cancer and emphasizes the mechanisms of action involved in the antiproliferative response in this type of tumor. 

## ***In vitro* studies about resveratrol effects on bladder cancer**

**Figure 1 -:**
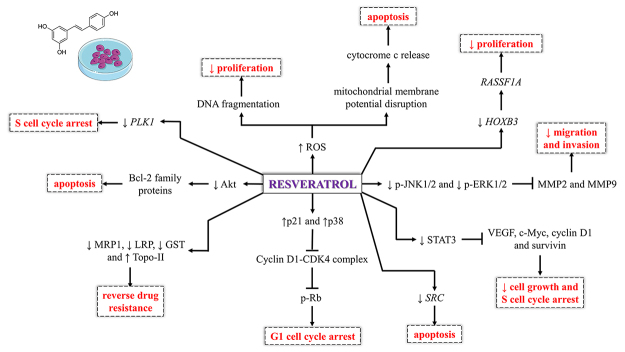
*In vitro* effects and mechanism of action of resveratrol. CDK4: cyclin-dependent kinase 4, GST: glutathione S‑transferase, *HOXB3*: homeobox B3, LRP: lung resistance protein, MMP2: matrix metalloproteinase 2, MMP9: matrix metalloproteinase 9, MRP1: multidrug resistance protein 1, p21: cyclin-dependent kinase inhibitor 1A, p38: p38 mitogen-activated protein kinase, p-ERK1/2: phosphorylated extracellular signal-regulated kinase 1 and 2, p-JNK1/2: phosphorylated c-Jun N-terminal kinase 1 and 2, *PLK1*: polo like kinase 1, p-Rb: phosphorylated retinoblastoma, *RASSF1A*: Ras association domain family member 1, ROS: reactive oxygen species, *SRC*: proto-oncogene tyrosine-protein kinase Src, STAT3: signal transducer and activator of transcription 3, Topo-II: topoisomerase II, VEGF: vascular endothelial growth factor.

The studies associating the effects of RSV and bladder cancer cells are summarized in Table 1 and Figure 1. Bai *et al.* (2010) conducted the first study showing the RSV effects in bladder cancer. The authors found that RSV caused G1 cell cycle arrest in T24 cells (transitional cell carcinoma), which was also found later by other authors in T24 and EJ cells (transitional cell carcinoma) (Yang *et al*., 2017). Bai *et al.* (2010) showed that the cell cycle arrest occurred through p21 and p38 activation. The increase of p21 and p38 expression inhibited Cyclin D1-CDK4 complex, an important mediator of G1-S transition that acts inhibiting Rb phosphorylation (Donjerkovic and Scott, 2000; Thornton and Rincon, 2009). The cell cycle arrest in T24 cells was accompanied by apoptosis through p-Akt inhibition. Akt signalling pathway is constitutively active in several types of human cancers, including bladder cancer (Sathe and Nawroth, 2018), and contribute to cancer progression, promoting cell proliferation and apoptosis suppression (Nitulescu *et al*., 2018). Decrease of p-Akt in T24 cells caused apoptosis through the mitochondrial pathways since there was modulation in Bcl-2 family proteins. 

**Table 1 -:** Effects of resveratrol in bladder cancer: *in vitro* studies.

Cancer cell	Concentration/time	Findings	Mechanisms	Reference
T24	50, 100, 150, 200, 250, 300 µM for 12, 24 or 48 h	Apoptosis Cell cycle arrest at G1 phase	↓p-AKT, ↓Bcl-2, ↓Bcl-xL, ↑Bax, ↓p-Bad, ↑cleaved caspase 3, ↑cleaved PARP ↑p21, ↑p-p38. ↓cyclin D1, ↓CDK4, ↓p-Rb	Bai *et al*., 2010
BTT739 and T24	12.5, 25, 50, 100 µM for 24 h or 50 µM for 6, 12, 24 or 48 h	Apoptosis	↑ROS production, mitochondrial membrane potential disruption, release of cytocrome c, ↑caspase 9, ↑caspase 3	Lin *et al*., 2012
ECV304 (derivation from T24)	0.1, 0.5, 1, 2.5, 5, 25, 50, 100 µM for 6 h 30 min or 50 µM for 12, 24 or 48 h	↑cell permeability and ↑DNA fragmentation Apoptosis	↑ROS production ↓Bad/Bcl-2 ratio	Stocco *et al*., 2012
EJ	100, 150, 200 µM for 1, 1.5 or 2 h in 24 h intervals during 72 h	↓cell growth and cell cycle arrest at S phase Apoptosis	↓STAT3, ↓p-STAT3, ↓p-STAT3 nuclear translocation, ↓c-Myc, ↓cyclin-D1, ↓survivin, ↓VEGF -	Wu *et al*., 2014
T24 and 5637	10, 30, 50 µM for 48 h.	Apoptosis	↓miR-21, ↓p-Akt, ↓Bcl-2	Zhou *et al*., 2014
T24	10, 25, 50, 100 µM for 6, 12 or 24 h	↓cell adhesion ↓cell migration and ↓cell invasion	- ↓p-JNK1/2, ↓p-ERK1/2, ↓MMP‑2, ↓MMP‑9	Bai *et al*., 2017
Pumc‑91/ADM	50, 100, 150, 200, 250, 300, 350 µM for 4, 48 or 72 h,	Sensitized Adriamycin-resistant cells Cell cycle arrest at S phase	↓MRP1, ↓LRP, ↓GST, ↑Topo-II -	Wang *et al*., 2017
T24 and EJ	20, 40, 60, 80, 100, 150, 200 µM for 6, 12, 24, 48 or 72h	Cell cycle arrest at G1 phase	-	Yang *et al*., 2017
RT4, 5637 and T24	12.5, 25, 50, 100, 150, 200, 250 µM for 24 h	↓cell proliferation ↓clonogenic survival Morphological changes Cell cycle arrest at phase S (5637 and T24) Apoptosis (RT4) Necrosis (T24) Antiproliferative effects	↑primary DNA damage ↓*PLK1* - ↓*PLK1* ↓*AKT*, ↓*mTOR*, ↓*SRC* - ↑*RASSF1A*/↓*HOXB3* (T24), ↓*DNMT1* (RT4)	Almeida *et al*., 2019
T24	25, 50, 75, 100, 125, 150, 200 µM for 6, 12, 24, 48 or 72 h	Apoptosis Morphological changes	- -	Yang *et al*., 2019

Likewise, Lin *et al.* (2012) found apoptosis through the intrinsic pathway in T24 and BTT739 cell lines (transitional cell carcinoma). RSV treatment caused disruption of mitochondrial membrane potential, which caused release of cytochrome c. In the cytosol, cytochrome c binds to Apaf-1, which recruits and activates caspase-9. This initiator caspase cleaves and activates effector caspases, mainly caspase-3, leading to the cell death (Elmore, 2007). It was also detected in T24 and BTT739 cells increase of reactive oxygen species (ROS) production after RSV treatment. The excessive ROS inside mitochondria might further induce oxidative modification of mitochondrial membrane lipids and change the permeability of the mitochondrial outer membrane, aggravating the disruption of mitochondrial membrane potential (Xu *et al*., 2010). Yang *et al.* (2019) also showed apoptosis in T24 cells after RSV treatment, but the authors did not discuss possible mechanisms.

In ECV304 cells (derivative of T24 cell line, transitional cell carcinoma), RSV treatment caused increase of cell permeability and DNA fragmentation, which was associated with ROS production (Stocco *et al*., 2012). ROS can react easily with nucleic acids, particularly DNA, triggering several structural changes including strand breakage (Bergamini *et al*., 2004). The study also found apoptosis accompanied by decrease of Bad/Bcl-2 ratio (pro-apoptotic/anti-apoptotic proteins) in ECV304 cells.

In EJ cells (transitional cell carcinoma), cell growth reduction, apoptosis and S phase cell cycle arrest after RSV treatment were accompanied by inhibition of STAT3 signaling pathway and nuclear translocations of Sirt1 and p53 (Wu *et al*., 2014). STAT3 acts as transcriptional regulator of a variety of tumor-promoting genes such as *VEGF*, c-*MYC*, *CCND1* (cyclin D1), *BIRC5* (survivin), which are involved in tumor development and progression (Santoni *et al*., 2015). Apoptosis might be associated with Sirt and p53 nuclear translocations. In cancer cells, Sirt1 is associated with cell death/survival and apoptosis by deacetylating of important transcriptional factors, including p53 (Shu *et al*., 2017). 

As mentioned previously, apoptosis caused by resveratrol have been related to Akt pathway (Bai *et al*., 2010). In T24 e 5637 cells (transitional cell carcinoma), the inhibition of Akt phosphorylation after RSV treatment occurred through inhibition of miR-21 expression (Zhou *et al*., 2014). Tao *et al.* (2011) showed the overexpression of miR‑21 promoted the proliferation of bladder cancer cell lines.

Metastasis is the most fatal characteristic of bladder cancer and it is a multistep process that is dependent on cellular activities, including migration and invasion of cancer cells (Steeg, 2006). Bai *et al.* (2017) focused on establishing the RSV inhibitory effects on these processes in T24 cells and found that the possible mechanism might be suppression of MAPK pathway. RSV treatment decreased JNK1/2 and ERK1/2 phosphorylation, resulting in the inhibition of metalloproteinases MMP‑2 and MMP‑9. Several studies have demonstrated that JNK1/2 and ERK1/2 transcriptionally regulate the expression of MMP‑2 and MMP‑9, which results in regulation of cell migration and invasion (Crowe *et al*., 2001; Wang *et al*., 2003; Moon *et al* 2004).

Wang *et al.* (2017) demonstrated that RSV treatment was able to reverse drug resistance in Adriamycin‑resistant pumc‑91 cells (Pumc‑91/ADM) (transitional cell carcinoma) through different mechanisms, as decrease of MRP1, LRP, GST and increase of Topo-II expression. All these proteins are important to drug resistance process. MRP1, multidrug resistance protein 1, acts as an efflux pump, which rapidly extrudes numerous anticancer drugs from the cancer cells (Lu *et al*., 2015). LRP, lung resistance protein, mediates drug resistance by transporting drugs from the nucleus to the cytoplasm through vesicular transport (Scheffer *et al*., 2000). GST, glutathione S‑transferase, is a phase II detoxification enzyme. However, tumor cells also utilize GST to form a complex between antitumor drugs and glutathione, which is excreted out of the tumor cell by Pgp and MRP (Dong *et al*., 2018). Topoisomerase II (Topo-II) is a nuclear protein that is usually highly expressed during active cell proliferation, being common its overexpression in tumors. However, it is supposed that decreased expression of Topo II is associated with drug resistance (Tsang *et al*., 2006; Yu *et al*., 2014). Several chemotherapeutic agents, as anthracyclines, epipodophy and amsacrine, interfere with DNA replication and promote DNA strand breaks via forming drug‑Topo‑II‑DNA complexes in cancer cells. The downregulation of Topo II may alter the crosslinking and production of DNA complexes, resulting in a decline in chemosensitivity (Zhao *et al*., 2016).

Almeida *et al.* (2019) showed that RSV has antiproliferative effects in bladder cancer cells independent of the *TP53* gene status (RT4 - *TP53* wild type, transitional cell carcinoma, 5637 and T24 - *TP53* mutant). *TP53* gene is considered the guardian of the genome, because it responds to stress signals inducing cell cycle arrest, apoptosis or DNA repair (Kastenhuber and Lowe, 2017). *TP53* mutations are common in muscle-invasive bladder cancer and are correlated with poor prognosis (Solomon and Hansel, 2016). In RT4, 5637 and T24 cells, the reduction of cell proliferation was associated with DNA primary damage caused by RSV treatment. The reduction of colonies formation was accompanied by reduction of *PLK1* gene expression after RSV treatment. Synthetic inhibitors of *PLK1* caused similar effect in the same cells (Brassesco *et al*., 2013), showing the importance of this gene for clonogenic survival.

The authors also demonstrated that different mechanisms of action can be activated in *TP53* mutated or wild type cells after RSV treatment (Almeida *et al*., 2019). In *TP53* mutated cells (5637 and T24), the decrease of *PLK1* expression was also associated with cell cycle arrest at S phase, since its encoded protein is necessary to S phase progress (Shen *et al*., 2013). In T24 cells, RSV treatment also caused modulation of pathways connected to *RASSF1A* and *HOXB3* genes. *RASSF1A* is a tumor suppressor gene, whose promoter region hypermethylation causes its inhibition in many cancers, including bladder cancer (Baylin and Herman, 2000). *RASSF1A* silencing occurs through the *HOXB3* oncogene that induces *DNMT3B* expression, a gene that encodes a DNA methylation enzyme. Overexpression of *DNMT3B* caused by *HOXB3* results in hypermethylation of *RASSF1A* promoter region (Palakurthy *et al*., 2009). 

In wild type cells (RT4), the apoptosis caused by RSV treatment was accompanied by reduction of *AKT/mTOR* and *SRC* gene expression (Almeida *et al*., 2019). Reduction of the protein encoded by *SRC* gene causes inhibition of FAK phosphorylation, an anti-apoptotic protein, favouring cell death (Kong *et al*., 2015). In this cell line, there was also reduction of *DNMT1* gene expression, which may be contributing to demethylation of tumor suppressor genes (Almeida *et al*., 2019).

The activity of RSV loaded in nanoformulation, aiming its use in blader cancer, was investigated only by Almeida *et al.* (2020). The authors showed that polymeric micelles were able to preserve the cytotoxic activity of free resveratrol in RT4 and T24 cells.

## ***In vivo* studies about resveratrol effects in bladder cancer**

**Figure 2 -:**
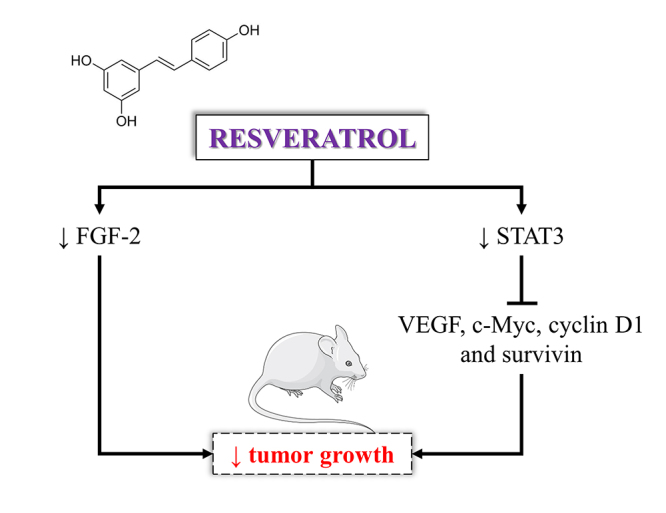
*In vivo* effects and mechanism of action of resveratrol. FGF2: fibroblast growth factor 2, STAT3: signal transducer and activator of transcription 3, VEGF: vascular endothelial growth factor.

Currently, there are only two studies about RSV in bladder cancer models (transitional cell carcinoma) *in vivo* (Table 2 and Figure 2). Bai *et al.* (2010) used a xenograft model of bladder cancer to investigate RSV effects *in vivo*. The authors found that RSV treatment significantly slowed the growth of tumors and it was associated with expression decrease of the pro-angiogenic regulators VEGF and FGF-2. Angiogenesis is an important process for tumor growth and progression, being an interest approach to treat cancer (Li T *et al*., 2018).

Wu *et al.* (2014) demonstrated that RSV intravesical treatment inhibited tumor growth in the orthotopic model used. The *in vivo* effect of RSV was associated with inhibition of STAT3 signalling pathway as discussed for its *in vitro* effects. Interestingly, the authors also showed that RSV treatment did not cause local irritation, indicating its safety for intravesical use. The transitional epithelia of bladder walls were undamaged, without capillary congestion or inflammatory lymphocyte infiltration. 

**Table 2 -:** Effects of resveratrol in bladder cancer: *in vivo* studies.

Animal model	Dose/duration	Findings	Mechanism	Reference
BALB⁄c-nude mice, male, 4 weeks old, injected subcutaneous with T24 cells into flanks	20 mg⁄ kg once daily for 4 weeks	↓tumor growth	↓VEGF, ↓FGF-2	Bai *et al*., 2009
BALB/c-nude mice, female, 4 weeks old, injected with EJ cells into sub-epithelial layer urinary bladders	200 µM in two day intervals for 28 days	↓tumor growth Apoptosis	↓STAT3, ↓p-STAT3, ↓c-Myc, ↓cyclinD1, ↓survivin, ↓VEGF -	Wu *et al*., 2014

## Studies about resveratrol effects in combination with other compounds in bladder cancer

The studies investigating the effects of RSV combined with other compounds to treat bladder cancer are summarized in Table 3. Alayev *et al.* (2016) studied the effects of RSV in combination with rapamycin on the inhibition of PI3K/Akt/mTOR signaling pathway. This pathway is related to the regulation of multiple cellular metabolic processes, cell growth, proliferation and survival (Yu and Cui, 2016). Its activation is very common in bladder cancer (Liu *et al*., 2018). Previous studies showed that rapamycin and everolimus (mTOR inhibitors) were able to inhibit growth of bladder tumor cells and bladder tumor xenograft models (Fechner *et al*., 2009; Mansure *et al*., 2009; Chiong *et al*., 2011). However, the use of mTOR inhibitors in monotherapy is not an interesting strategy. mTOR is involved in a negative feedback loop with PI3K and Akt; when mTOR levels decline, PI3K and Akt levels increase and cross-talk with other growth pathways (Abdelnour-Berchtold *et al*., 2010). In this context, Alayev *et al.* (2016) demonstrated that RSV and rapamycin combination was effective since it inhibited the levels of several mTOR downstream effectors (p-56K1, p-S6, p-4EBP1, and p-eIF4B) as well as was able to prevent rapamycin-induced reactivation of Akt. The authors also showed that the combination caused apoptosis, reduced cell migration and clonogenic survival.

One of the options to address multidrug resistance problem is using drug combination (Lou *et al*., 2018). It has been reported that RSV can reverse multidrug resistance in cancer cells. Moreover, it can sensitize cancer cells to standard chemotherapeutic agents when used in combination with clinically used drugs (Ko *et al*., 2017). Cho *et al.* (2019; 2020) studied the effects of RSV to overcome gemcitabine resistance in bladder cancer. The authors showed that the combination of RSV with gemcitabine caused an additive cytotoxic effect in bladder cancer cells T24-GCB (gemcitabine resistant cell line). They investigated modulation in some proteins related to drug resistance in bladder cancer, as ATP binding cassette subfamily C member 2 (ABCC2), deoxycytidine kinase (DCK), thymidine kinase 1 (TK1), and thymidine kinase 2 (TK2). However, RSV may act by other mechanism since those proteins levels did not change as expected.

**Table 3 -:** Resveratrol effects in combination with other compounds in bladder cancer.

Cancer cell	Concentration/time	Combination	Findings	Mechanisms	Reference
HCV39, 639V and MGH-U1	100 µM for 24 or 48 h	Rapamycin 20 nM	Antiproliferative effects Apoptosis ↓cell migration ↓clonogenic survival	↓p-Akt, ↓p-mTOR, ↓p-56K1, ↓p-S6, ↓p-4EBP1, ↓p-eIF4B ↑cleaved caspase 3, ↑cleaved PARP, ↓survivin, ↓mcl1 - -	Alayev *et al*., 2016
T24-GCB	75 and150 µM for 72 h	Gemcitabine 10 µM	Sensitized gemcitabine-resistant cells Apoptosis	- ↑cleaved PARP	Cho *et al*., 2019
T24-GCB	75 and 150 µM for 72 h	Gemcitabine 10 µM	Sensitized gemcitabine-resistant cells	-	Cho *et al*., 2020

## Future perspectives

Clinical trials with healthy volunteers have shown that RSV administration does not cause serious adverse events (Boocock *et al*., 2007; Almeida *et al*., 2009; Brown *et al*., 2010). However, these studies also showed that RSV presents rapid metabolism and low bioavailability, requiring strategies to improve its future use. Some strategies such as optimization of drug delivery with formulations and synergistic or additive interactions with other phytochemicals were reported to increase RVS bioavailability (Amri *et al*., 2012; Smoliga and Blanchard, 2014; Santos *et al*., 2019).

Although there are no studies about RSV and bladder cancer in humans, the effects of this compound in other cancers were already investigated in clinical trials. Patel *et al.* (2010) demonstrated that the treatment with RSV (0.5-1.0 g/day, for 8 days) reduced cell proliferation in colorectal tumor samples from patients. Additionally, the authors reported good tolerability of patients to treatment. Howells *et al.* (2011) also demonstrated good results in patients with colorectal cancer and liver metastasis. After the treatment with RSV (5 grams/day for 14 days), there was an increase in caspase-3 expression in liver tumor samples. Another study showed that the intake of 5-50 mg, twice daily, for 12 weeks caused a reduction of methylation of the tumor suppressor gene *RASSF1A* in women at increased risk of breast cancer (Zhu *et al*., 2012). These clinical findings, the possibility of optimization of drug delivery, few side effects observed and the results of in vivo observations and in vitro experiments discussed above are optimistic and encourage further studies about RSV effects in bladder cancer.

## Conclusion

RSV has been found to inhibit cancer cell proliferation, cell migration, and invasion, induce cell cycle arrest, and trigger apoptosis in bladder cancer cells. Besides that, RSV decrease tumor growth in bladder cancer models *in vivo*. These anticancer effects are related to its ability to modulate several signaling molecules involved in cancer processes. Thus, RSV is a potential agent for treating bladder cancer. Further *in vivo* studies using the compound alone or in combination with other drugs are needed to confirm the effectiveness of RSV in bladder cancer.
